# Alimentação de Idosos Diabéticos e não Diabéticos no Brasil

**DOI:** 10.36660/abc.20211001

**Published:** 2022-02-14

**Authors:** Mariana de Souza Dorna

**Affiliations:** 1 Universidade Estadual Paulista Júlio de Mesquita Filho São Paulo SP Brasil Universidade Estadual Paulista Júlio de Mesquita Filho – Medicina Interna,São Paulo, SP - Brasil

**Keywords:** Alimentação, Idoso, População Idosa, Diabetes Mellitus, Sedentarismo, Epidemiologia, Dieta do Mediterrâneo, Fatores de Risco, Frutas, Verduras

Com o crescimento da população e, consequentemente, da população idosa, aumentou também a preocupação com a saúde mental e qualidade de vida desses indivíduos. Dados da Federação Internacional de Diabetes (IDF) sugerem que, em 2019, aproximadamente 463 milhões de adultos (20-79 anos) possuem o diagnóstico de diabetes (DM). Dentre eles, 1 em cada 5 são idosos.^1 - 3^ Ainda segundo o IDF, o Brasil ocupava a 5ª posição em relação aos números de pessoas diagnosticadas com DM.

Estilo de vida sedentário e dieta desbalanceada estão entre os principais fatores para o desenvolvimento do diabetes. Ainda assim, estudos epidemiológicos, que buscam associações entre consumo alimentar e DM, são limitados.^4^ Por outro lado, padrões alimentares com base em baixo consumo de alimentos ultraprocessados e maior ingestão de frutas e vegetais frescos mostram-se eficazes tanto na prevenção quanto no manejo de doenças crônicas não transmissíveis (DCNT).^5^ Esse padrão dietético denominado “Mediterrâneo” ganha espaço por promover hábitos de vida saudáveis e respeito à sazonalidade dos alimentos.^6^

O Estudo Longitudinal de Saúde do Adulto (ELSA-Brasil) evidenciou resultados, relacionados ao alto consumo de carnes processadas, que apontam o aumento do risco de resistência insulínica e elevação de 40% do risco de desenvolvimento de diabetes em homens.^7^ Ainda assim, estudos epidemiológicos sobre consumo alimentar são limitados, e muitas vezes os resultados, poucos consistentes.^4 , 8^

Um dos desafios para realização desses estudos no Brasil, é a grande extensão territorial do país, associada a heterogeneidade populacional e diferenças culturais e alimentares regionais. Alguns estudos são baseados em dados compilados por pesquisas como a Pesquisa de Orçamento Familiar (POF), o Inquérito Nacional de Alimentação e o sistema de Vigilância de Fatores de Risco e Proteção para as Doenças Crônicas por Inquérito Telefônico (VIGITEL).^4 , 9 , 10^

Nesta edição, Francisco et al.,^11^ utilizaram o Vigitel 2016 como ferramenta de mapeamento do consumo alimentar de idosos diabéticos e não diabéticos residentes em capitais brasileiras e no Distrito Federal (4). Esse estudo apresenta um expressivo tamanho amostral, com pouco mais de 13 mil idosos entrevistados e prevalência de aproximadamente 27% de diabetes. Entre o grupo dos idosos com DM foi observada maior frequência no consumo de hortaliças cruas, importante por causa do aumento da ingestão de fibras alimentares e seu conhecido papel na manutenção da glicemia. Contudo, nesse mesmo grupo, contatou-se maior consumo de carnes com gordura aparente bem como de doces e bebidas adoçadas, o que não apenas dificulta a manutenção da glicemia como pode aumentar o risco cardiovascular desses indivíduos.

O estudo traz à luz as dificuldades metodológicas para realização de inquéritos alimentares populacionais no Brasil. Ainda que os inquéritos telefônicos gerem dados confiáveis, o trabalho apresenta viés de seleção, como apontado pelos autores, por incluírem apenas indivíduos com telefonia fixa. Esse fator evidencia uma das grandes dificuldades em trabalhos populacionais em um país com dimensões continentais. Além disso, o recorte transversal do trabalho permite uma visão global acerca da frequência alimentar dessa população, mas não evidencia, contudo, o caminho a ser percorrido no que tange à acessibilidade alimentar segura e de qualidade para a população. Assim sendo, o estudo não permite estabelecer relações entre consumo alimentar e adesão a orientações nutricionais e o diagnóstico da DM. Por fim, são justamente as dificuldades desse estudo que o tornam extremamente relevante no campo de estudo de hábitos alimentares populacionais.
