# Supplementary material for: Alimentação de Idosos Diabéticos e não Diabéticos no Brasil
Source: Arq Bras Cardiol. 2022 Feb 14;118(2):398–9. [Article in Portuguese] doi: 10.36660/abc.20211001 (PMC8856694; doi:10.36660/abc.20211001)
Supplement: Translation

# Translation: Food Intake among the Diabetic and Non-Diabetic Elderly Population in Brazil

**Authors:** Mariana de Souza Dorna

**Affiliations:** 1 Brazil Universidade Estadual Paulista Júlio de Mesquita Filho – Medicina Interna, São Paulo, SP - Brazil

**Keywords:** Feeding, Aged, Elderly Population, Diabetes Mellitus, Sedentarism, Epidemiology, Diet Mediterranean, Risk Factors, Fruits, Vegetables

With the growth in the general population and, consequently, in the elderly population, concerns about mental health and the quality of life of these individuals have also risen. Data from the International Diabetes Federation (IDF) suggest that, in 2019, approximately 463 million adults (20-79 years of age) have received a diabetes mellitus (DM) diagnosis. Among them, 1 in every 5 are elderly individuals.^1 - 3^ According to the IDF, Brazil ranks 5^th^in relation to the number of people diagnosed with DM.

A sedentary lifestyle and an unbalanced diet are among the main factors for the development of DM. Nonetheless, epidemiological studies, which search for associations between food consumption and DM, are limited.^4^ By contrast, eating standards based on the consumption of ultraprocessed foods and the greater intake of fresh fruits and vegetables has proven to be effective in both the prevention and management of chronic non-communicable diseases (NCDs).^5^ This diet standard, known as “Mediterranean”, is gaining ground, as it promotes healthy lifestyles and a high regard for seasonal foods.^6^

The Brazilian Longitudinal Study of Adult Health (ELSA-Brasil) has shown results related to the high consumption of processed meats, which point to an increased risk for insulin resistance and a 40% higher risk of developing DM in men.^7^ However, epidemiological studies about food consumption are scarce in the literature, and their results are often highly inconsistent.^4 , 8^

One of the challenges to conduct these studies in Brazil is the large territorial size of the country, associated with the population’s heterogeneity, as well as its differences in culture and regional eating habits. Some studies are based on data compiled by specific research projects, such as the Household Budget Survey (HBS), the National Diet and Nutrition Survey, and the Surveillance System of Risk and Protection Factors for Chronic Diseases by Telephone Inquiry (VIGITEL).^4 , 9 , 10^

In this edition, Francisco et al.^11^ used Vigitel 2016 as a tool to map the food consumption of the diabetic and non-diabetic elderly population in Brazilian capitals and the Federal District of Brasilia (4). This study presents an expressive sample size, with a little over 13,000 interviews conducted with elderly individuals. Results showed a prevalence of approximately 27% of the individuals diagnosed with DM. In the group of elderly individuals with DM, what was found was a greater frequency of the consumption of raw vegetables, an important cause for the rise in the intake of fibers and their well-known role in the maintenance of blood glucose levels. However, in this same group, what was also found was a greater consumption of fatty meats, as well as sweets and sugary drinks, which not only hinder the maintenance of one’s blood glucose levels, but also increase the cardiovascular risks in these individuals.

This study sheds light on the methodological difficulties to conduct population eating habit surveys in Brazil. Even though telephone inquiries generate reliable data, the data present selection bias, as pointed out by the authors, since only individuals with in-house telephones were included. This factor reveals one of the major difficulties in population studies in a continental-sized country. Moreover, the cross-sectional cut of the study enables a broader overview of the eating habits of this population; however, it does not show the path to be taken regarding a safe and high-quality food accessibility for the population. Therefore, this study does not allow one to establish connections between food consumption and adherence to nutritional advice and the diagnosis of DM. Finally, it is exactly the difficulties encountered in this study that make it extremely relevant to the field of study concerning population eating habits.
